# Coping strategies in obsessive-compulsive patients during Covid-19 lockdown

**DOI:** 10.1016/j.ijchp.2021.100223

**Published:** 2021-01-16

**Authors:** Ángel Rosa-Alcázar, María Dolores García-Hernández, José Luis Parada-Navas, Pablo J. Olivares-Olivares, Sergio Martínez-Murillo, Ana I. Rosa-Alcázar

**Affiliations:** a Department of Psychology, Catholic University of Murcia, Spain; b Department of Personality, Assessment & Psychological Treatment, University of Murcia, Spain; c Faculty of Education, University of Murcia, Spain

**Keywords:** Obsessive-compulsive disorder, Coping strategies, COVID-19, Lockdown, Experiment, Trastorno obsesivo-compulsivo, Estrategias de afrontamiento, COVID-19, Confinamiento, Experimento

## Abstract

The main aim of this study was to compare coping strategies in obsessive-compulsive disorder (OCD) patients and a healthy control group during COVID-19 lockdown and to analyze the relationship with some variables which may influence results (depression, anxiety, comorbidity, subtype of obsession-compulsion). Method: There were 237 participants, 122 OCD and 115 healthy controls, aged 17-61 years old (*M* = 33.48, *SD* = 11.13). Results: Groups showed differences in the use of some adaptive strategies (positive reinterpretation, acceptance, humor) and maladaptive (denial, self-blame). Within obsessive-compulsive group, comorbidity affected the greater use of inappropriate strategies (denial, substance abuse and self-blame) while type of obsession-compulsion did not influence use. Anxiety and depression levels were related to the use of less adaptive strategies. Conclusions: These findings strengthen the need for training in the use of effective and adaptive coping strategies, making it necessary to improve clinical follow-up of these patients. It is relevant to be in contact with healthcare professionals, review medication and observe the anxiety and depression levels.

## Antecedentes/Objetivo

El objetivo de este estudio fue comparar las estrategias de afrontamiento utilizadas por pacientes con Trastorno obsesivo-compulsivo (TOC) durante el confinamiento debido a la COVID-19 frente a controles sanos y analizar su relación con algunas variables que pueden influir en los resultados (ansiedad, depresión, comorbilidad, tipo de obsesiones-compulsiones). Método: Los participantes fueron 237, 122 diagnosticados de Trastorno obsesivo-compulsivo y 115 controles sanos, con edades comprendidas entre 13 y 58 años (M = 34,60, DT = 10,41). Resultados: Los grupos presentaron diferencias en el uso de algunas estrategias adaptativas (reinterpretación positiva, aceptación, humor) y desadaptativas (negación, autoculpa). Dentro del grupo TOC, la comorbilidad afectó para el mayor uso de estrategias inadecuadas (negación, abuso de sustancias y autoculpa), mientras que el tipo de obsesiones-compulsiones no influyó en el uso de las mismas. Los niveles de ansiedad y depresión estaban relacionadas con el uso de estrategias menos adaptativas. Conclusiones: Estos hallazgos refuerzan la necesidad de entrenar en la utilización de estrategias de afrontamiento eficaces y adaptativas, siendo necesario mejorar el seguimiento clínico de estos pacientes. Es importante estar en contacto con profesionales de la salud, revisar la medicación y observar los niveles de ansiedad y depresión.

The Coronavirus disease 2019 (COVID-19) will have an impact on various spheres of life such as the economy, human health, health care, politics, etc. The World Health Organization has also reported the impact of the pandemic on mental health and psycho-social consequences. Quarantine has affected people’s usual activities, routines, and livelihoods and this may lead to an increase in loneliness, anxiety, depression, insomnia and harmful alcohol and drug use (World Health Organization, 2020).

The psychological repercussions in both general and clinical populations is causing serious mental health problems, highlighting severe anxiety and depressive symptoms, severe stress levels, posttraumatic stress, etc. (Brailovskaia and Margraf, 2020, Bueno-Notivol et al., 2021, Inchausti et al., 2020, Li et al., 2020, Mazza et al., 2020, Pfefferbaum and North, 2020, Xiang et al., 2020).

Fear of disease and contagion together with prevention and safety behaviors, such as hand washing, physical distancing, avoidance of stimuli and situations, hypervigilance to somatic sensations, economic problems, reductions in social interaction may increase the risk of serious and disabling mental health conditions, such as anxiety disorders, post-traumatic stress disorder, obsessive- compulsive disorder (OCD) (Fiorillo and Gorwood, 2020, Fontenelle and Miguel, 2020, Pozza et al., 2020, Silva et al., 2020). The uncontrollable and unpredictable nature of COVID-19 has likely led to extraordinary stress in the general population. Previous studies have reported that controllable or uncontrollable stressful experiences may provoke or exacerbate psychological and physical pathologies (Matheson and Anisman, 2003, Slavich, 2016). It is important to take into account variables both of the stressor itself (frequency, duration, type, number, control) and the individual's abilities to cope (Monroe et al., 2007). Focusing on OCD, the elements of containment of the pandemic will produce reinforcement of obsessive thinking and compulsive behavior and exacerbation of symptoms (Fineberg et al., 2020, Rivera and Carballea, 2020, Silva et al., 2020). These symptoms have been reported following previous epidemics such as SARS, MERS, etc. (Kumar & Somani, 2020). The increase in symptoms might not be immediate and depends on various factors such as the need to keep hands clean each time a person comes from outside, the constant checking for information from various media sources about the virus, the stocking up of masks, soaps, sanitizers and disinfectants all of which can lead to hoarding and panic shopping, etc. (Banerjee, 2020).

The COVID-19 pandemic is also causing an increase in obsession and compulsion severity. Some variables have been associated with more elevated worsening, such as presenting contamination symptoms, poor personal hygiene and higher severity (Davide et al., 2020). Benatti et al. (2020) have reported a significant emergence of new obsessions and compulsions, increased suicidal ideation, Internet checking, sleep disturbances, avoidance behaviors, and work difficulties.

One factor that plays a significant role in the psychological and psychosocial control is coping strategies. Lazarus and Folkman (1986) defined these “coping strategies” as constantly changing cognitive and behavioral efforts, which are developed to handle specific demands that are valued as situations that exceed a person's resources. Coping modulates the differences that exist between individuals in stressful situations, as a result of trying to change these situations or regulate emotional reactions (Morán et al., 2010).

Ravindran et al. (2002) showed that depression has been associated with excessive use of emotion-focused coping, particularly emotional containment and rumination. Ladouceur et al. (2000) carried out a study analyzing different coping strategies in OCD patients versus anxious and healthy participants. Results showed that the clinical groups used a greater number of strategies but did so ineffectively. The OCD group reported a significantly higher proportion of strategies specifically linked to the thought content, highlighting such things as convincing oneself, distracting activity, seeking reassurance, saying stop, analyzing. The OCD group presented excessive importance of thoughts and hyper-responsibility. Moritz et al. (2018) also found that adaptive coping in OCD patients was worse relative to healthy controls and to clinical controls with depression. Moreover, the depressive group presented greater acceptance, reevaluation of problems as opportunities and keeping one’s cool, while the OCD group showed greater catastrophizing. Recently, Damirchi et al. (2020) in a community sample found a significant negative relationship between self-talk and emotional coping style, death anxiety, and obsessive-compulsive disorder during COVID-19.

We have not found any study analyzing coping strategies in OCD patients during COVID-19. It is important to know what have been used by OCD patients during COVID-19 lockdown and their relationship with anxiety and depression level, both to study their status and to be able to use prevention and treatment strategies.

Therefore, the aim of the present study was to examine differences in coping strategies in OCD patients and a healthy control group in relation to COVID-19. Specifically, the study aimed to: (1) study coping strategies in OCD patients and a healthy control group during lockdown; (2) examine coping strategies controlling level of anxiety and depression; (3) assess differences in coping strategies in the OCD group due to comorbidity and OCD subtypes; (4) assess the relationship between coping strategies and anxiety and depression levels in the OCD group.

## Method

### Participants

There were 237 participants aged between 13-58 years (*M* = 34.60, S*D* = 10.41) diagnosed with OCD (American Psychiatric Association APA, 2013) and a healthy control group. Women comprised 55.70% of the sample. Primary obsessions before COVID-19 were aggressive (21.30%), miscellaneous (18.10%) and somatic (14.80%). Primary compulsions were checking (41.80%), cleaning/washing (23.80%), and miscellaneous (17.20%). During COVID-19 lockdown, 36.80% showed new obsessions related to contamination (42.30%), somatic (33.30%), aggressive (14.30%), religious (6.10%), dysmorphic (4%), accumulation, sexual and symmetric/ordering (2%). The new compulsions were cleaning/washing (57.80%), checking (11.10%), ordering (8.90%), repeating (11.10%), hoarding (6.70%) and miscellaneous (4.40%). The average duration of OCD was 10.06 years (*SD* = 10.79). 36.10% patients suffer comorbidity. 67% OCD patients received pharmacological treatments (antidepressant = 69.50%, antipsychotic + antidepressant = 30.50%) and 100% were under psychological treatment.

Inclusion criteria for participants in the clinical group included a diagnosis of OCD according to DSM criteria. The Control group (CG) could not present any current psychopathological disorder. All participants had to be in lockdown due to COVID-19 (April 2020).

Participants in the clinical group were excluded if they presented a current psychopathology with Bipolar Disorder, Schizophrenic Spectrum Disorders and other Psychotic Disorders, Personality Disorders, Anorexia, Bulimia, disorders related to substance and addictive dependence and Neurocognitive Disorders. Sample characteristics are presented in Table 1.

**Table 1:** Sample characteristics.

Characteristics	OCD (*n* = 122)	CG (*n* = 115)	*t/χ^2^*
Age (*M ± SD*)	34.50 ± 10.34	34.71 ± 10.53	n.s.
Sex *n* (%)			n.s.
Men	66 (54.10)	66 (57.40)	
Women	56 (45.90)	49 (42.60)	
Years OCD duration (Mean ± SD)	16.06 ± 10.70	-	
Comorbidity n (%)		-	
No comorbidity	82 (67.20)		
Comorbidity	40 (32.80)		
Type of comorbidity *n* (%)		-	
Depression	14 (35)		
Disorders personality	5 (12.50)		
Disorders estrés Trauma	1 (2.50)		
Others problems Anxiety (Social anxiety, agoraphobia, panic)	20 (50)		
Marital status *n (%)*			n.s.
Single	65 (53.30)	65 (56.60)	
Married	48 (39.30)	47 (40.90)	
Divorced	9 (7.40)	3 (2.70)	
Educational level *n* (%)			*X*^2^ (3) = 17.09; *p* < .001
Elementary	7 (5.70)	1 (0.90)	
Secundary education	23 (18.09)	7 (6.10)	
High school	31 (25.40)	24 (20.80)	
University student	61 (50)	83 (72.20)	
Who did you spend quarantine with? (%)			n.s.
Alone	12 (9.80)	7 (6.10)	
Friends/Partner/Flatmate	3 (2.5)	5 (4.30%)	
Family	107 (87.70)	103 (89.60)	
Anxiety (*M ± SD*)	11.71 ± 4.45	7.42 ± 4.02	*t*(235) = 7.78; *p* < .001
Depression (*M ± SD*)	8.08 ± 4.49	5.31 ± 3.54	*t*(235) = 5.39; *p* < .001

*Note*. OCD: Obsessive compulsive disorder; CG: Control group. *M* = mean; *SD*: Standard deviation; n.s.: Not significant.

### Procedure

**Figure 1:**
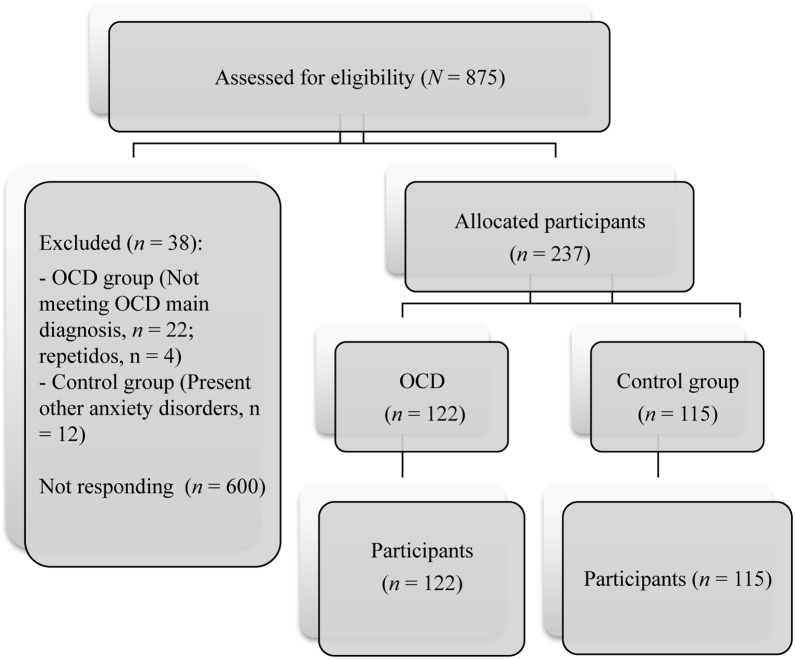
CONSORT Flow diagrams of study development.

The study met the ethical standards of the Declaration of Helsinki and has been approved by the Ethics Committee of the University of Murcia (Spain). All families provided written informed consent. The sample was recruited from two contexts: clinical and community. Once clinical group was formed, the non-clinical group was recruited in order to be equal in age and sex. The procedure was as follows: a) Contact some associations/public and private clinics/university workers of Murcia (Spain, April 2020), and b) complete the anonymous online survey during 20 minutes. Responses were saved on a secured server at the Universtiy of Murcia.The test presentation order was the same for all participants. Participation was voluntary and free. Recruitment is shown in Figure 1.

### Measures

Yale Brown Obsessive Compulsive Scale (Y-BOCS; Goodman et al., 1989). Comprising 10 items assessing OCD severity. It contains two subscales, Obsessions (range = 0-20) and Compulsions (range = 0-20) and a Total score (range = 0-40). The scale has a high internal consistency (α = .87-.90), and good convergent validity (*r* = .74 - *r* = .47). A total average greater than or equal to 16 is considered of clinical significance. Evaluation was carried out by the psychologists who treated the patients, reporting data before and during the lockdown. OCD patients also completed this instrument, with the percentage of agreement being very high regarding the type of obsessions and compulsions (97% and 98, respectively). The severity data were taken from those reported by clinicians before and during lockdown. Cronbach's alpha in this study was .87 (clinician) and .86 (participants OCD). This instrument was only applied to clinical participants.

Hospital Anxiety and Depression Scale (HADS; Zigmond & Snaith, 1983). Self-report measure of anxiety and depression of 14 items rated on a 4 point Likert scale (0 a 3). It was divided into Anxiety (HADS-A) and Depression (HADS-D) subscales both containing seven items. Cronbach's alpha in this study was .77 and .79 (depression non-clinical and clinical group), .75 and .77 (anxiety non-clinical and clinical group), .82 and .84 (total non-clinical and clinical group).

Spanish Version of the Brief COPE (COPE-28; Morán et al., 2010). 28 items and 14 subscales answered on a Likert-type ordinal scale of 4 response alternatives (from 0 to 3). Coping dimensions are: (1) Active coping (initiate direct actions, increase your own efforts, eliminate or reduce the stressor); (2) Planning (thinking about how to deal with the stressor and planning action strategies); (3) Instrumental support (seeking help, advice, and information from competent people); (4) Use of emotional support (receiving emotional support of sympathy and understanding); (5) Self-distraction (concentrating on other projects, trying to distract oneself with other activities, trying not to concentrate on the stressor); (6) Relief (increased awareness of one's emotional distress, accompanied by a tendency to express or discharge those feelings); (7) Behavioral disconnect (reduce efforts to deal with the stressor, including giving up effort to achieve goals with which the stressor interferes); (8) Positive reframing (looking for the positive and favorable side of the problem and trying to improve or grow from the situation); (9) Denial (denying the reality of the stressful event); (10) Acceptance (accepting what is happening, that it is real); (11) Religion (tendency to return to religion in times of stress, increase participation in religious activities); (12) Substance use -alcohol, medications (drinking alcohol or other substances in order to feel good or to help cope with the stressor); (13) Humor- (making jokes about the stressor or laughing at stressful situations); and (14) Self-blame (criticizing and blaming oneself for what happened). Second order factors are: Cognitive coping, Social coping, Avoidance coping and Spiritual coping. Higher scores reflect a higher tendency to implement the corresponding coping strategies. We asked participants to report how often they used the strategy described in each project to respond to COVID-19. Cronbach's alpha in this study was .80 and .82 (clinical and non-clinical group, respectively).

### Data analysis

Firstly, Chi-square and one-factor ANOVA were used to examine potential group differences in clinical and demographic variables. Subsequently, *t* Student, ANOVA test and post-hoc comparisons (Tukey or Games-Howel) were carried out. A covariance analysis was performed when there were significant differences between groups in some variables (anxiety and depression) considered influential in their coping strategies. Independent samples tests (Kruskal Wallis H test) were performed within the clinical group, taking into account presence/absence of comorbidity and OCD subtypes. The Pearson correlation was used to analyze the relationship between variables. Cohen's d (standardized mean differences) were calculated to estimate the magnitude of between-group differences, 0.20 low, 0.50 medium, and 0.80 high. All participants were included in analyzes. SPSS Statistic 22.00 was used for statistical analysis.

## Results

### Group equivalence

Groups were equivalent in sex (*p* = .610), age (*p* =  .875), who they live with (*p* =  .313), and marital status (*p* = .127). Differences were seen in educational level (*p* =  .001), anxiety (*p* <  .001) and depression (*p* <  .001). The OCD group achieved worse results in the clinical variables mentioned. See Table 1.

### A comparison of OCD and healthy CG in coping strategies

The *t* Student showed a significant group effect in the following positive strategies: positive reframing (*p* <  .001), acceptance (*p* <  .001), humor (*p* < .001), CG obtaining best scores. The OCD group achieved better results in instrumental support (*p* =  .030) and religion (*p* <  .001). In addition, the clinical group showed higher means in denial (*p* =  .004) and self-blame (*p* <  .001). Second order factors, cognitive coping and spiritual coping showed statistical significance (*p* <  .001), the CG used cognitive strategies most, while the OCD group presented emotional strategies most. Higher ES were found in acceptance (*d* = 0.84) and humor (*d* = 0.68). ES in cognitive coping was medium. See Table 2.

**Table 2:** Student *t* in coping strategies.

VD	Group OCD (*n* = 122) *M ± SD*	Group Control (*n* = 115) *M ± SD*	*t*	*p*	Cohen´s d*
Active coping	3.39 ± 1.50	3.67 ± 1.61	-1.32	.185	-0.19
Planning	3.07 ± 1.74	3.38 ± 1.54	-1.44	.150	-0.18
Instrumental support	2.85 ± 1.85	2.35 ± 1.62	2.18	.030	0.27
Positive reframing	2.66 ± 1.61	3.43 ± 6.50	-3.59	< .001	-0.47
Acceptance	3.58 ± 1.61	4.93 ± 1.09	-7.50	< .001	-0.84

Emotional support	3.44 ± 1.82	3.28 ± 1.74	0.71	.479	0.09
Denial	1.55 ± 1.76	0.94 ± 1.42	2.90	.004	0.35
Relief	2.62 ± 1.71	2.31 ± 1.58	1.45	.149	0.18
Self-blame	2.57 ± 1.69	1.63 ± 1.24	4.79	< .001	0.55
Humor	2.15 ± 1.74	3.34 ± 1.70	-5.32	< .001	-0.68
Religion	1.95 ± 1.98	0.98 ± 1.57	4.15	< .001	0.49
Self-distraction	3.88 ± 1.57	3.75 ± 1.54	0.64	.524	0.08
Substance use	0.63 ± 1.34	0.45 ± 1.18	1.09	.278	0.13
Behavioral disconnet	2.02 ± 1.21	1.92 ± 1.13	0.62	.53	0.10
	*Coping second level*				
Cognitive coping	14.68 ± 5.09	17.29±4.59	-4.13	*<* .001	-0.52
Social coping	8.92 ± 4.49	7.95 ± 4.08	1.74	.084	0.22
Avoidance coping	10.80±4.43	10.12±3.40	1.32	.189	0.15
Spiritual coping	1.95 ± 1.98	0.98 ± 1.57	4.15	< .001	0.49

*Note*. OCD: Obsessive compulsive disorder; CG: Control group. ES = Effect size Cohen* Negative indicated that the group compared in first place reached worst score acieved by the group appearing in second place.

### Coping strategies controlling level of anxiety and depression

As anxiety and depression variables reached statistically significant differences between groups, covariance analysis was conducted. It was observed that when controlling anxiety and depression, planning showed significant differences, *p* =  .017, this strategy being higher in the CG. However, there were no differences in the use of instrumental support (*p* =  .912).

### Intragroup comparisons based on comorbidity and OCD subtypes

As for comorbidity, differences were observed in denial (*p* =  .026), substance use (*p* =  .022), self-blame (*p* =  .002) and avoidance coping (*p* =  .001). Participants who did not suffer comorbidity with other disorders achieved lower scores in maladaptative strategies.

As regards the obsession type variable, no statistically significant differences were found in any analyzed variables (*p* >  .05). Additionally, types of compulsions showed no differences in any strategy analyzed.

### Correlation between coping strategies and anxiety and depression in OCD group

Results showed a significant positive correlation among anxiety and emotional strategies, such as relief, denial and self-blame; a negative correlation was found with acceptance. In addition, depression was negatively correlated with active coping, positive reframing, and humor. Cognitive coping was negatively related with depression. See Table 3.

**Table 3:** Correlations among anxiety and depression and coping strategies.

	Anxiety	Depression
Active coping	.11	-.28**
Plannig	.22*	-.05
Instrumental support	.30**	.127
Positive refraiming	-.10	-.38***
Acceptance	-.39***	-.57***
Emotional support	.15	-.04
Denial	.43***	.32***
Relief	.27**	.12
Self-blame	.40***	.30**
Humor	-.15	-.33**
Religion	.26**	.13
Self-distraction	.07	-.18*
Substance use	.11	.23*
Behavioral disconnet	.17	.19*
Coping second level		
Cognitive coping	-.01	-.36***
Social coping	.28**	.08
Avoidance coping	.32***	.11
Spiritual coping	.26**	.13

***p <  .001, **p <  .01; *p < .05.

## Discussion

The general aim of this study was to know the coping strategies of Spanish OCD patients during the COVID-19 lockdown (April 2020). The coping strategies most used by both groups were acceptance, use of emotional support, self-distraction, active coping and planning. These are positive coping strategies that helped control stress produced at the beginning of lockdown. It is important to note that these data were collected during the month of April 2020. At that time, the population was not yet subjected to prolonged stress, something that is happening at the present time.

The comparison regarding the use of coping strategies between groups, indicated that OCD patients presented higher means in instrumental support (seeking help, advice, and information from competent people ), religion (tendency to return to religion in times of stress, increase participation in religious activities), denial (denying the reality of the stressful event) and self-blame (criticizing and blaming oneself for what happened). These may be related to the obsessions and compulsions themselves, specifically, with blame obsessions, checking and religious compulsions. In addition, seeking help, advice, and information in excess can be counterproductive for these patients, and can exacerbate obsessional and anxiety behaviors, in some cases becoming verification compulsions (Finerberg et al., 2020). Some patients spend hours each day watching television and checking online media reports of COVID-19, increasing levels of discomfort and worsening OCD symptoms (French & Lyne, 2020). On the other hand, the healthy control group used positive coping strategies: reframing (looking for the positive and favorable side of the problem and trying to improve or grow from the situation), acceptance (accepting what is happening, that it is real) and humor (making jokes about the stressor or laughing at stressful situations), strategies that allow facing the stressor perceived as non-controllable in a more positive way.

As indicated by Matheson and Anisman (2003), given that coping strategies likely operate in conjunction with one another, their effectiveness may vary as a function of alternative strategies that are adopted concurrently or sequentially. In this case, the strategies used by the CG are along the lines of facing the situation more positively and decisively.

This coincides with results obtained by Ladouceur et al. (2000) finding that OCD patients reported strategies linked to thought content, those most used being seeking, analyzing and distracting activity. Moritz et al. (2018) also found that OCD and depressive patients displayed more maladaptive coping and avoidance as well as less adaptive coping than nonclinical controls.

Another element to take into account is whether those strategies used by the OCD group are related to the neutralization of obsessions, it being necessary not to reinforce this mechanism (Freeston & Ladouceur, 1997). Trying not to think (denial) may create conditions for intrusive thoughts that culminate in rumination (Wegner, 1989).

The next target was to learn whether coping strategies can be influenced by anxiety and depression. It was observed that, when controlling anxiety and depression, planning strategy was significantly higher in the healthy control group. In addition, there were no differences in the use of instrumental support between both groups. Therefore, these clinical variables seem to be relevant in which strategies are used (Rosa-Alcázar et al., 2019, Seçer and Ulaş, 2020, Storch et al., 2010).

OCD is also associated with other psychological disorders, such as tics, attention deficit-hyperactivity disorder, autism spectrum disorders (ASD) depression and anxiety disorders which increase degree of discomfort and complicate both treatment and prognosis (Storch et al., 2010). For this reason, we analyzed whether there were differences in coping strategies in the OCD group due to comorbidity. Comorbidity showed differences in denial, substance use, self-blame and avoidance coping. OCD patients who did not suffer comorbidity with other disorders achieved lower scores in maladaptive strategies. Therefore, the greater the comorbidity, the greater the severity and use of maladaptive strategies. This result is in line with that reported by other research where comorbidity was associated with poor response to treatment, and therefore worse use of strategies to cope with stressful situations (Barrett et al., 2005).

On the other hand, obsession and compulsion types did not affect coping strategies used. This obtained result may be unusual, since as mentioned above, the coping strategies used by the OCD group seemed to be related to obsessions and compulsions. We would have expected patients with contamination, somatic obsessions and cleaning/washing and accumulation compulsions, to present more maladaptive strategies (Banerjee, 2020, French and Lyne, 2020). However, given these results, we must take three elements into account. The first is that perhaps, the moment data was collected, the first weeks of confinement, when stress is not so acute, affects results, since; the situation of maintained stress is what often worsens the patient´s condition. Therefore, it is necessary to analyze results over time. The second element to consider is that the OCD patient worries excessively by overestimating real threats, such as with COVID-19, or incorporating unreal threats. It may occur that OCD patients worsen or even change obsessions and compulsions, regardless of whether these are related to the variables enhanced by the pandemic (Fineberg et al., 2020, Rivera and Carballea, 2020). The third element is that we have analyzed the main subtypes reported by patients and therapists, but have not evaluated the dimensions of the symptoms. Perhaps if we had been able to carry out a face-to-face evaluation we could have analyzed these dimensions, being able to arrive at more conclusive results.

The final aim was to assess the relationship between coping strategies and anxiety and depression in the OCD group. Higher anxiety was related to relief, denial, religion, self-blame and spiritual coping. Therefore, higher depression is negatively related with active coping, positive reframing, acceptance, self-distraction and humor. These results are in line with previous studies where high depression and anxiety levels enhance coping that do not lead to effectively resolving stressful situations (Matheson & Anisman, 2003). The high correlation between anxiety and depression and denial and self-blame might enhance rumination, the main response of the disorder we are studying. Therefore, we can indicate that changes in coping strategies depend on affective states (Lazarus, 2000).

The current study has important implications for clinical practice in OCD patients. Firstly, coping strategies in OCD patients and CG are more focused on emotions than problem solving. For this reason, and as the pandemic might take time to disappear, it is important to train in useful and adaptive coping for both populations. Teaching a flexible repertoire of coping strategies will help people cope with different stressors and in a range of circumstances. This requires providing psychoeducation on COVID-19 and its impact on physical and psychological health, distinguishing between these rational adaptive rituals and obsessional and compulsive acts in the context of OCD, controlling exposure and consumption of the internet and mass media (Rosa-Alcázar et al., 2020). Governments should study if the information provided generates panic or whether it promotes self-protection behaviors. The consumption of excessive media coverage pertinent to COVID19 may impact negatively on mental health (Balasubramanian et al., 2020). In addition, it would be important to establish a daily routine, even if stuck at home. Patients under quarantine or staying at home under restrictions are at great risk of sleep problems, thus increasing anxiety and worsening OCD symptoms. It is relevant to be in contact with professionals, review medication and observe the anxiety and depression levels that increase the use of maladaptive strategies in OCD patients (Fineberg et al., 2020, Rosa-Alcázar et al., 2019).

This study had some limitations. First, selection of patients was not random. This was a cross sectional study. Participants were assessed by a self-report scale. The application of the questionnaire was online, so that administration conditions and situational variables that could influence completion and responses could not be controlled. In addition, due to lockdown, administration possibilities were very limited.

As future prospects, it would be relevant to evaluate two uncontrolled variables: tolerance to uncertainty (a variable that is being studied in patients with OCD, and very relevant to the current situation with COVID-19), and whether they themselves or their relatives have been infected with COVID-19. Furthermore, it would be interesting to carry out a longitudinal research, which studies how participants evolve with this pandemic over time, in particular at 6, 12, and 18 months. The course of the epidemic along with the presence of other stressors (unemployment, family illness, financial problems) could influence changes in people's coping strategies and challenge emotions.

## References

[bib0005] American Psychiatric Association APA (2013). Manual diagnóstico y estadístico de los trastornos mentales (DSM-5).

[bib0010] Balasubramanian A., Paleri V., Bennett R., Paleri V. (2020). Impact of COVID-19 on the mental health of surgeons and coping strategies. Head & Neck.

[bib0015] Banerjee D. (2020). The COVID-19 outbreak: Crucial role the psychiatrists can play. Asian Journal of Psychiatry.

[bib0020] Barrett P., Farrell L., Dadds M., Boulter N. (2005). Cognitive-behavioral family treatment of childhood obsessive-compulsive disorder: Long-term follow-up and predictors of outcome. Journal American Academic Child Adolescent Psychiatry.

[bib0025] Benatti B., Albert U., Maina G., Fiorillo A., Celebre L., Girone N., Fineberg N., Bramante S., Rigardetto S., Dell’Osso B. (2020). What Happened to Patients With Obsessive Compulsive Disorder During the COVID-19 Pandemic? A Multicentre Report From Tertiary Clinics in Northern Italy. Frontiers in Psychiatry.

[bib0030] Brailovskaia J., Margraf J. (2020). Predicting adaptive and maladaptive responses to the Coronavirus (COVID-19) outbreak: A prospective longitudinal study. International Journal of Clinical and Health Psychology.

[bib0035] Bueno-Notivol J., Gracia-García P., Olaya B., Lasheras I., López-Antón R., Santabárbara J. (2021). Prevalence of depression during the COVID-19 outbreak: A meta-analysis of community-based studies. International Journal of Clinical and Health Psychology.

[bib0040] Damirchi E.S., Mojarrad A., Pireinaladin S., Grjibovski A.M. (2020). The Role of Self-Talk in Predicting Death Anxiety, Obsessive-Compulsive Disorder, and Coping Strategies in the Face of Coronavirus Disease (COVID-19). Iranian Journal of Psychiatry.

[bib0045] Davide P., Andrea P., Martina O., Andrea E., Davide D., Mario A. (2020). The impact of the COVID-19 pandemic on patients with OCD: Effects of contamination symptoms and remission state before the quarantine in a preliminary naturalistic study. Psychiatry Research.

[bib0050] Fineberg N.A., Van Ameringen M., Drummond L., Hollander E., Stein D.J., Geller D., Walitza S., Pallanti S., Pellegrini L., Zohar J., Rodríguez C., Menchon J., Morgado P., Mpavaenda D., Fontenelle L., Feusner J., Grassi G., Lochner C., Veltman D., Dell´Osso B. (2020). How to manage obsessive-compulsive disorder (OCD) under COVID-19: A clinician’s guide from the International College of Obsessive Compulsive Spectrum Disorders (ICOCS) and the Obsessive-Compulsive Research Network (OCRN) of the European College of Neuropsychopharmacology. Comprehensive Psychiatry.

[bib0055] Fiorillo A., Gorwood P. (2020). The consequences of the COVID-19 pandemic on mental health and implications for clinical practice. European Psychiatry.

[bib0060] Fontenelle L.F., Miguel E.C. (2020). The impact of COVID‐19 in the diagnosis and treatment of obsessive-compulsive disorder. Depression and Anxiety.

[bib0065] Freeston M.H., Ladouceur R. (1997). What do patients do with their obsessive thoughts?. Behaviour Research and Therapy.

[bib0070] French I., Lyne J. (2020). Acute exacerbation of OCD symptoms precipitated by media reports of COVID-19. Irish Journal of Psychological Medicine.

[bib0075] Goodman W.K., Price L.H., Rasmussen S.A., Mazure C., Fleischmann R.L., Hill C.L., Heninger G.R., Charney D.S. (1989). The Yale-Brown obsessive compulsive scale: I. Development, use, and reliability. Archives of General Psychiatry.

[bib0080] Inchausti F., García-Poveda N.V., Prado-Abril J., Sánchez-Reales S. (2020). La psicología clínica ante la pandemia COVID-19 en España. Clínica y Salud.

[bib0085] Kumar A., Somani A. (2020). Dealing with Corona virus anxiety and OCD. Asian Journal of Psychiatry.

[bib0090] Ladouceur R., Freeston M.H., Rhéaume J., Dugas M.J., Gagnon F., Thibodeau N., Fournier S. (2000). Strategies used with intrusive thoughts: A comparison of OCD patients with anxious and community controls. Journal of Abnormal Psychology.

[bib0095] Lazarus R.S. (2000). Toward better research on stress and coping. American Psychological Association.

[bib0100] Lazarus R.S., Folkman S. (1986). Estrés y procesos cognitivos.

[bib0105] Li W., Yang Y., Liu Z.H., Zhao Y.J., Zhang Q., Zhang L., Xiang Y.T. (2020). Progression of Mental Health Services during the COVID-19 Outbreak in China. International Journal Biological Sciences.

[bib0110] Matheson K., Anisman H. (2003). Systems of coping associated with dysphoria, anxiety and depressive illness: A multivariate profile perspective. Stress.

[bib0115] Mazza C., Ricci E., Biondi S., Colasanti M., Ferracuti S., Napoli C., Roma P. (2020). A Nationwide Survey of Psychological Distress among Italian People during the COVID-19 Pandemic: Immediate Psychological Responses and Associated Factors. International Journal of Environmental Research and Public Health.

[bib0120] Monroe S.M., Slavich G.M., Torres L.D., Gotlib I.H. (2007). Severe life events predict specific patterns of change in cognitive biases in major depression. Psychological Medicine.

[bib0125] Morán C., Landero R., González (2010). COPE-28: un análisis psicométrico de la versión en español del Brief COPE. Universitas Psychologica.

[bib0130] Moritz S., Fink J., Miegel F., Nitsche K., Kraft V., Tonn P., Jelinek L. (2018). Obsessive-compulsive disorder is characterized by a lack of adaptive coping rather than an excess of maladaptive coping. Cognitive Therapy and Research.

[bib0135] Pfefferbaum B., North C.S. (2020). Mental health and the Covid-19 pandemic. New England Journal of Medicine.

[bib0140] Pozza A., Mucci F., Marazziti D. (2020). Risk for pathological contamination fears at coronavirus time: Proposal of early intervention and prevention strategies. Clinical Neuropsychiatry.

[bib0145] Ravindran A.V., Matheson K., Griffiths J., Merali Z., Anisman H. (2002). Stress, coping, uplifts, and quality of life in subtypes of depression: a conceptual model and emerging data. Journal Affective Disorders.

[bib0150] Rivera R.M., Carballea D. (2020). Coronavirus: A trigger for OCD and illness anxiety disorder?. Psychological Trauma: Theory, Research, Practice, and Policy.

[bib0155] Rosa-Alcázar Á., Olivares-Olivares P.J., Martínez-Esparza I.C., Parada-Navas J.L., Rosa-Alcázar A.I., Olivares-Rodríguez J. (2020). Cognitive flexibility and response inhibition in patients with Obsessive-Compulsive Disorder and Generalized Anxiety Disorder. International Journal of Clinical and Health Psychology.

[bib0160] Rosa-Alcázar Á., Rosa-Alcázar A.I., Olivares-Olivares P.J., Parada-Navas J.L., Rosa-Alcázar E., Sánchez-Meca J. (2019). Family involvement and treatment for young children with Obsessive-Compulsive Disorder: Randomized control study. International Journal of Clinical and Health Psychology.

[bib0165] Seçer İ., Ulaş S. (2020). An Investigation of the Effect of COVID-19 on OCD in Youth in the Context of Emotional Reactivity, Experiential Avoidance, Depression and Anxiety. International Journal of Mental Health and Addiction.

[bib0170] Silva R.M., Shavitt R.G., Costa D.L. (2020). Obsessive-compulsive disorder during the COVID-19 pandemic. Brazilian Journal of Psychiatry.

[bib0175] Slavich G.M. (2016). Life stress and health: A review of conceptual issues and recent findings. Teaching of Psychology.

[bib0180] Storch E.A., Lewin A.B., Geffken G.R., Morgan J.R., Murphy T.K. (2010). The role of comorbid disruptive behavior in the clinical expression of pediatric obsessive-compulsive disorder. Behaviour Research and Therapy.

[bib0185] Wegner D.M. (1989). White bears and other unwanted thoughts: Suppression, obsession, and the psychology of mental control.

[bib0190] World Health Organization (2020). Mental health and COVID-19.

[bib0195] Xiang Y.T., Yang Y., Li W., Zhang L., Zhang Q., Cheung T., Ng C.H. (2020). Timely mental health care for the 2019 novel coronavirus outbreak is urgently needed. The Lancet Psychiatry.

[bib0200] Zigmond A.S., Snaith R.P. (1983). The Hospital Anxiety and Depression Scale. Acta Psychiatrica Scandinavica.

